# Ecologically relevant levels of multiple, common marine stressors suggest antagonistic effects

**DOI:** 10.1038/s41598-017-06373-y

**Published:** 2017-07-24

**Authors:** Rolanda Lange, Dustin Marshall

**Affiliations:** Centre for Geometric Biology/School of Biological Sciences, Monash University, Clayton, VIC 3800 Australia

## Abstract

Stressors associated with global change will be experienced simultaneously and may act synergistically, so attempts to estimate the capacity of marine systems to cope with global change requires a multi-stressor approach. Because recent evidence suggests that stressor effects can be context-dependent, estimates of how stressors are experienced in ecologically realistic settings will be particularly valuable. To enhance our understanding of the interplay between environmental effects and the impact of multiple stressors from both natural and anthropogenic sources, we conducted a field experiment. We explored the impact of multiple, functionally varied stressors from both natural and anthropogenic sources experienced during early life history in a common sessile marine invertebrate, *Bugula neritina*. Natural spatial environmental variation induced differences in conspecific densities, allowing us to test for density-driven context-dependence of stressor effects. We indeed found density-dependent effects. Under high conspecific density, individual survival increased, which offset part of the negative effects of experiencing stressors. Experiencing multiple stressors early in life history translated to a decreased survival in the field, albeit the effects were not as drastic as we expected: our results are congruent with antagonistic stressor effects. We speculate that when individual stressors are more subtle, stressor synergies become less common.

## Introduction

Global changes, such as anthropogenic climate change, environmental pollution, or trophic shifts in communities, challenge our marine environments by novel stressors or unprecedented stressor levels^1, 2^. Determining the ecological effects of anthropogenic stressors and the potential for resistance or resilience enables more informed environmental management decisions. Anthropogenic stressors often occur in combination^3, 4^. For example, in heavily modified coastal systems, organisms will experience both water pollution and competition from invasive species. Although organisms can be resilient to a single stressor, their probability of coping with additional stressor can decrease, especially if the stresses act synergistically^4, 5^. This means that multiple stressors can have larger impacts than the sum of individual stressors (and *vice versa* for stressor antagonism). Therefore, studies involving only single stressors may over- or underestimate resilience if stressors act synergistically or antagonistically.

Multistressor approaches can aid a better understanding of adaptation in complex environments and have sky-rocketed during the past two decades. This is likely driven by an urgency to predict and potentially mitigate climate change, which often triggers multiple stressors^3, 6^. Stressors that are hypothesized to increase due to global change have been particularly well-studied: many studies have investigated the combined effects of temperature, salinity, acidification, UV, hypoxia, metal pollution stress, and their effects have been investigated in formal meta-analyses^7, 8^. Importantly, not all of these environmental variables are always experienced as stressors: temperature, salinity and UV are variables that are inherent abiotic components of marine environments. Most benthic marine invertebrates are adapted to commonly experienced ranges of these variables, for instance temperature can also increase fitness when experienced within optimum ranges^9, 10^. These common environmental variables only become stressors when experienced within pessimum ranges where energy is required to maintain metabolic functions^11^. Pessimum thresholds can also depend on energy budget, such that stressors are more likely to be experienced as such under limited energy supply^12^.

Recent expansions in this research area have increased our understanding of multiple stressors in marine organisms and ecosystems, particularly in benthic marine invertebrates^5, 8, 13, 14^. Several patterns have arisen from single studies and meta-analyses, though these are not universal. One emerging pattern is the vulnerability of early life history stages, such as gametes or embryonic and larval stages, compared to later adult stages^7, 13, 15–17^. Yet, depending on phylum and stressor tested, very early embryonic stages are sometimes more resilient than later embryonic stages. For example, in echinoderms early embryos appear more resilient to acidification and temperature stress than late embryos^18, 19^, and this has been attributed to more protective compounds at early stages^20^. Perhaps unsurprisingly, calcifying invertebrates appear most vulnerable to temperature and acidification stress^7, 14, 21, 22^, but this pattern also appears not universal^23–25^. Responses can also depend on parental pre-exposure (adaptive transgenerational plasticity) or previous acclimation to stressors, individuals that had been pre-exposed to various stressors tend to be more resilient^8, 26–28^. Sessile species have less opportunity to behaviorally avoid stressors, and tropical species tend to occur closer to their critical thermal limits, which may make such species more vulnerable to change^9, 29, 30^. Moreover, the timing of stressor exposure likely influences outcomes: simultaneous stressor exposure is most common to test stressor effects and hypothesized to increase the likelihood of finding synergistic effects. In the field, however, exposure timing might be much more complex and not captured by simultaneous stressor studies only^8^.

Stressor interactions also depend on the stressors under investigation. Salinity and temperature has been the most commonly studied stressor pair in studies on marine invertebrates, with Crain *et al*.^5^ detecting mostly antagonistic interactions on stressor responses, and Przeslawski *et al*.^7^ detecting synergistic interactions. Perhaps their different findings where shaped by including studies focusing on different life history stages and phyla. Under salinity or temperature stress, the toxicity of pollutants increases, and this is an almost universal pattern^31^. For example, in an oyster^32^, a polychaete^33^ and a crab^34^, copper and low salinity had synergistic effects in embryos. Most likely, synergism is caused by osmoregulation being negatively affected by metals, and rising temperature increasing metabolic activity and metal solubility and thus uptake of toxic metals^31, 32^. While stressor responses appear to be highly plastic and context-dependent^5, 7, 8, 35^, synergism appears to become the prevalent response when more than two stressors are tested^5^.

Following the establishment of baseline impacts of multiple stressors on marine invertebrates and realization of how variable stressor responses can be, Przeslawski *et al*.^7^ made recommendations to advance the field, for instance by (i) testing more subtle stressor levels in experiments and to mimic levels as they are already found in the field, (ii) incorporating a wider variety of stressors, with inclusion of local anthropogenic stressors and natural stressors (a combination of two of three common stressors, pH, salinity or temperature being the current norm), and (iii) testing the effects of multiple stressors in the field. The latter is because stressor effects can be highly context-dependent - environmental complexity can have major impacts on stressor outcomes^36, 37^. For instance, under ample nutrient supply most organisms are better equipped to cope with stressors^38–40^. Przeslawski *et al*.^7^ recommendations aim to increase the ecological realism of multifactorial stressor studies.

To further our understanding of multiple stressors as they are experienced in the field, we tested the effects of multiple stressors from both anthropogenic and natural sources in a field experiment. Specifically, we wanted to infer how the common sessile marine invertebrate *Bugula neritina* (henceforth *Bugula*) is affected by functionally varied and ecologically realistic levels of stressors, experienced during early life history. We used stressor levels as they already occur in more contaminated sites (with *Bugula* populations) or during more extreme weather events, and predicted that multiple stressors would affect these organisms disproportionately strongly – especially as it seems that synergism increases when more than two stressors are tested^5^. We tested the effects of copper pollution, low salinity, increased temperature and increased larval duration. Copper pollution is a common local anthropogenic stressor and is toxic, particularly to early life history stages^31, 41–44^. While salinity and temperature fluctuations are naturally occurring phenomena, these global stressors have increased under climate change^1^. The fourth stressor we included is one that *Bugula*, and most other marine invertebrates, face early in their life history: time spent finding a suitable habitat as a larva. At least half of coastal marine invertebrates, including *Bugula*, have a non-feeding larval stage^45^, which elevates the energetic costs of dispersal: prolonged larval durations reduce survival and adult fitness^46–48^. We either allowed larvae to settle immediately or prolonged the larval phase experimentally. We then exposed *Bugula* to stressors treatments during early post-settlement, and then transplanted individuals into the field. There, they experienced spatial environmental variation – an almost inherent feature of field experiments – including differences in conspecific densities. This natural variation allowed us to test for density driven context-dependence of stressor effects.

## Methods

### Study species and collection

*Bugula neritina* is a colonial, cheilostome bryozoan often found as part of the fouling community. Colonies are arborescent and consist of 100s to 1000s of zooids, which filter-feed by extending their lophophores (a tentacle with 15–20 arms) into the water column. Each zooid is a separate, clonal individual connected to the colony by pores that extend to the above and below neighbouring zooids. Colonies are founded by single settlers, which grow into colonies by budding zooids. Bifurcating branches form at regular intervals. Within few weeks of settlement, colonies gain reproductive maturity and each zooid can potentially produce one egg in an externally visible brood chamber^49^. For each of four experimental blocks we collected at least ten reproductively mature *Bugula* colonies from pontoons located at Altona pier, Victoria, Australia (37°52′22.96″S, 144°49′48.91″E) and transported them to Monash University, where they were kept in in complete darkness for 48 h. We then spawned colonies using standard methods^49^. In brief, colonies were induced to spawn by exposing them to bright light and larvae were settled by pipetting them on to biofilmed, roughened acetate sheets.

### Stressor treatments

One of our objectives was to only subject individuals to ecologically realistic levels found in the field for each of the individual stressors. Importantly, our chosen abiotic stressors do co-occur. Heavy rainfall (salinity drops) is most likely in summer (increased temperatures) and associated with increased pollutants from stormwater drainages and urban river runoffs^50, 51^. For dissolved copper, levels measured in Port Phillip Bay are in the range of 47 and 130 μg/L^52^, depending on distance to river runoffs. Salinity after heavy rainfall can drop to 29 psu^52^. Water temperatures range from 10 °C to 22 °C, but have recently climbed above 25 °C during the 2015/2016 El Nino (www.baywx.com.au). Larval durations vary between minutes and 16 h, but given suitable substrate the majority of *Bugula* larvae will have settled within 2 hrs^49^. With this prior information, we set the stress treatments as follows: larval duration prolonged by 2 hrs, copper exposure at 65 μg/L, salinity at 30 psu, and temperature at 22 °C. In contrast, we set the control treatment as follows: larval duration not prolonged, no exposure to any additional copper other than trace amounts naturally occurring in seawater (see ref. 26 for copper manipulation methods), salinity at 36 psu, and temperature at 17.5 °C. These conditions contribute to optimal growth (refs 27, 49 and 53, unpublished data). The configuration of stressor treatments is laid out in Table 1. Depending on which stressor was manipulated we will refer to treatments as the control; single stressor treatments: delay treatment, salinity treatment, heat treatment, copper treatment, and the multiple stressors treatment.

**Table 1:** Stressor manipulations: how the six stressor treatments were configured.

Treatment (overall)	Control	Delay	Salinity	Heat	Copper	Multiple
Larval treatment	no delay	2 h delay	no delay	no delay	no delay	2 h delay
Juvenile treatment	36 psu + 17.5 °C + 0 µg/L Cu	36 psu + 17.5 °C + 0 µg/L Cu	30 psu + 17.5 °C + 0 µg/L Cu	36 psu + 22 °C + 0 µg/L Cu	36 psu + 17.5 °C + 65 µg/L Cu	30 psu + 22 °C + 65 µg/L Cu

### Experimental design

After spawning, one part of the spawned larvae was allocated to a delay treatment (multiple-stressors or delay-stressor only, see Table 1), where we delayed settlement using standard methods^54^. In brief, larvae were delayed from settling by constant, gentle water movement. Once settlement was delayed for two hours, larvae were given the opportunity to settle. All other larvae could settle immediately (see Table 1 for treatment design and timing). We induced settlement by placing a larva in a drop of seawater on biofilmed, pre-roughened acetate sheets. Larvae that had not settled within two hours were gently rinsed off. Thereafter, all acetate sheets were cut into 1 cm × 1 cm squares. These small acetate squares, each containing one settler, were then allocated to one of six treatments. Settlers that had been delayed for two hours were randomly allocated to either the multiple stressor or the delay stressor treatment groups (cf. Table 1 for differences between treatments). All other settlers were randomly allocated to the control, copper, salinity, or heat treatment groups (cf. Table 1).

Settlers were kept in milipore-filtered seawater, and each larvae was kept in a 2 ml well of a 24-well plate in a controlled temperature cabinet for 24 h.

After 24 h under these controlled treatment conditions we released settlers into the field. To achieve this, we glued the acetate sheets with the settlers on to 11 cm × 11 cm acrylic plates (48 plates in total). Each plate contained one replicate per treatment (6 acetate sheets with individual settlers, constituting 48 replicates per treatment per block). These plates were transported to the Blairgowrie Yacht Squadron (38°21′20.16″S, 144°46′22.82″E). Blairgowrie Yacht Squadron experiences strong, unidirectional water flow and is located in a relatively high water quality area (http://www.epa.vic.gov.au), such that the background level of stressors they experienced there can be assumed to be medium to low and is representative for the overall region of Port Phillip Bay. There, they were attached to the bottom surface of one of four 60 cm × 60 cm panels, which horizontally hung 1.5 m off a floating pontoon fastened by acrylic rope. Each panel carried 12 plates. We returned every two weeks to record growth and survival of each individual for four weeks. We repeated this procedure four times, with192 total replicates per treatment. Due to a heatwave that occurred during our experiment, only colonies in one out of our four blocks survived to produce offspring, so we have relatively few fecundity measurements.

### Statistical analysis

All statistical analyses were done using R version 3.0.2^55^, using packages ggplot2^56^, lme4^57^, and plyr^58^. We analysed effects of stressor treatments and conspecific density (survivors per plate, excluding the focal) on our response variables survival, colony size, and log fecundity (only colonies in block 4 survived to reproductive maturity due to seasonal variation) using generalised linear models (survival with a binomial error distribution and colony size with a poisson error distribution) and multiple regression (fecundity, with a gaussian error distribution) with treatment and panel as fully crossed fixed effects, and treatment and density as fully crossed fixed effects. Note that panel was treated as a fixed factor because there were only three levels per block; below five to six levels random effects cannot be tested as such^59^. To test the statistical significance of interactions we used log-likelihood ratio tests to compare models with all fixed effects interactions against models that omitted each interaction. Non-significant interaction terms were dropped from our models^60^. To test the overall statistical significance of fixed effects we used Wald tests. We visually assessed our final models for homogeneity and normality of error variances^60^. We then extracted our survival model coefficients to analyse whether additive, antagonistic or synergistic effects of multiple stressors were most likely by comparing the effects of single and multiple stressors on the probability of survival, relative to the probability of survival under the control treatment (as % survival relative to control), as is common^35, 61^. We converted log -likelihood treatment effect coefficients into the probability of survival. Because we found significant conspecific density effects we also modeled the probability of survival under different densities.

We want to stress that our experimental design was not orthogonal. A fully factorial would have required 16 treatment groups, which was not feasible for our field set up and would have strongly diminished our statistical power. Moreover, the resulting 2-, 3-, and 4-way interactions would have been beyond our abilities to interpret. The difficulties and redundancies in analyzing full factorial designs that move beyond 3 × 3 layouts are widely acknowledged in the statistical community in general^62^, in the research of multiple stressor effects in specific^5, 7^, and has inspired use of alternative experimental designs for the analysis of >2 stressors^63, 64^. Instead of testing for stressor interactions by using a full factorial design and ANOVA, we compared single stressor and multiple stressor effects with a null model under multiplicative effects. Multiplicative null models are often chosen when it can be assumed that individuals who have been killed by one stressor cannot be killed by another^61^. In brief, we used the single stressor treatment coefficients to calculate a null expectation of multiple stressor effects under multiplicative stressor effects and compared these with the multiple stressor treatment coefficient. Using the method described in ref. 61 we calculated our multiplicative null expectation by multiplying survival under each stressor relative to the control. In case standard errors of the multiple stressor treatment crossed the null expectation, we refuted stressor synergism or antagonism. A multiple stressor effect lower than the null expectation we categorized as congruent with stressor antagonism, larger than null expectation effects as congruent with stressor synergism.

## Results

**Figure 1:**
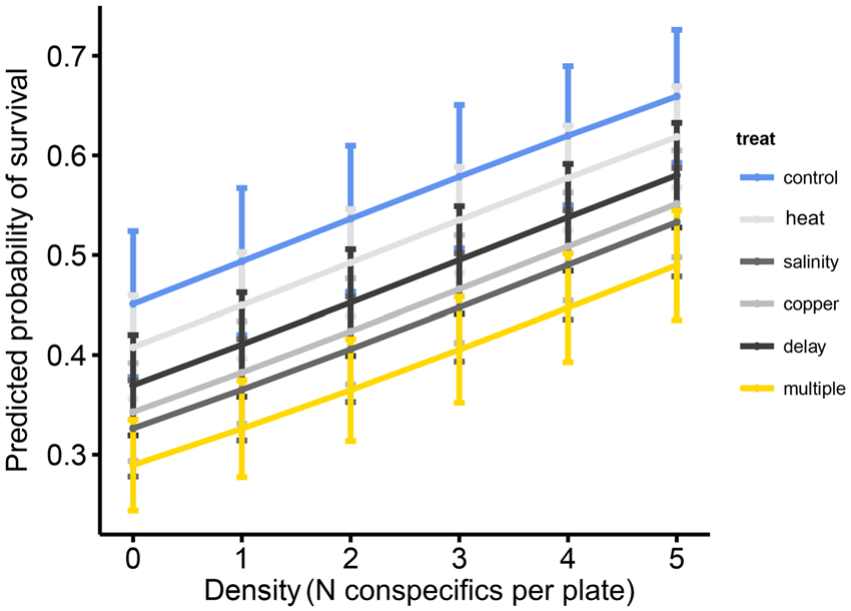
Extracted and transformed logistic regression model coefficients (with standard errors) predicting treatment (line colours) and conspecific density effects (x-axis as number of surviving conspecifics per plate) on the probability of survival (y-axis).

Stressor treatments affected colony survival (Fig. 1, Table 2): colonies that experienced control conditions during early development survived best, whereas those that experienced single stressors had slightly lower survival, and those that experienced multiple stressors had the lowest survival (Table 3). Conspecific density also affected survival in that when more conspecifics were present, survival was higher (Fig. 1, Tables 2 and 3). When analyzing the coefficients gained from our analysis of survival, our expected multiplicative null model response was lower than the decrease in survival found in the multiple stressor treatment. Survival under single stressors relative to survival under control conditions were: salinity = 77% ± 9%, heat = 92% ± 12%, copper = 81% ± 9%, delay = 86% ± 9%. Survival under multiple stressors was 70% ± 9%. This suggests antagonistic effects of multiple stressors under ecologically relevant levels of stress under a multiplicative null model (multiplicative null model expectation = 77% × 8% × 81% × 86% = 49% survival).

**Table 2:** Wald test results testing the effects of stressor treatment, panel, and density of conspecifics on survival, fecundity, and size.

	*df*	Χ^2^	*P*
*Dependent variable: survival*			
Treatment	5	12.7	**0.026**
Panel	15	51.2	**<0.001**
Density	1	8.9	**0.003**
*Dependent variable: fecundity*			
Treatment	5	11.4	**0.043**
Panel	3	22.1	**<0.001**
Density	1	0.37	0.54
*Dependent variable: size*			
Treatment	5	3.4	**0.043**
Panel	15	309.1	**<0.001**
Density	1	3.6	0.059

P-values below 0.05 are highlighted by bold lettering.

**Table 3:** Treatment effects on survival and fecundity from multiple logistic regression and GLM.

	Coefficients (survival: log odds)	*SE*	*P*
*Dependent variable: survival*			
Intercept	−0.2	0.3	0.52
Heat	−0.18	0.22	0.42
Salinity	−0.53	0.22	**0**.**02**
Copper	−0.45	0.22	**0**.**04**
Delay	−0.34	0.22	0.12
Multiple	−0.7	0.22	**0**.**002**
Conspecific density	0.17	0.06	**0**.**002**
*Dependent variable: fecundity*			
Intercept	4.39	0.90	<**0**.**001**
Heat	1.86	0.86	**0**.**03**
Salinity	1.62	0.92	0.08
Copper	1.72	1.00	0.09
Delay	−0.42	0.89	0.64
Multiple	0.86	0.94	0.36

P-values below 0.05 are highlighted by bold lettering.

**Figure 2:**
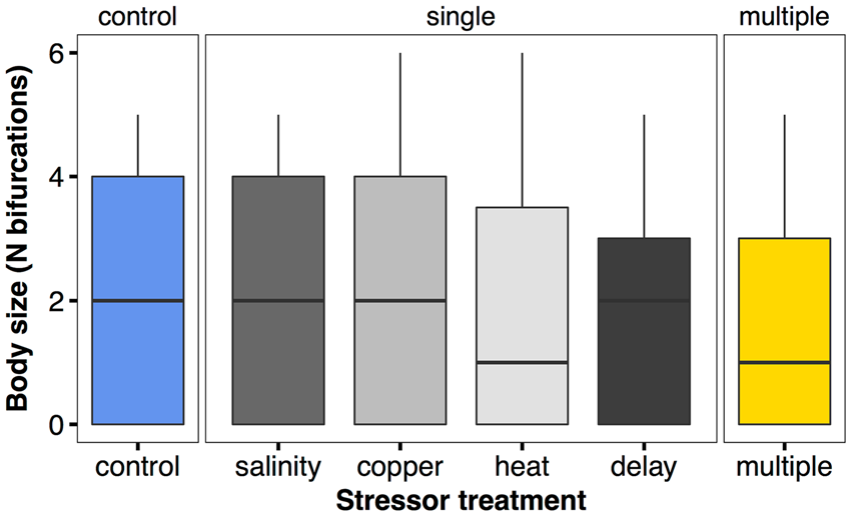
Comparison of control (blue), single stressor (grey), and multiple stressor (yellow) effects on colony size, measured as the number of bifurcations. Boxplots from raw data, treatment effects on x-axis, colony size on y-axis.

Our analysis of mean colony size revealed that this parameter was unaffected by stressors experienced during early development (Fig. 2, Table 2).

**Figure 3:**
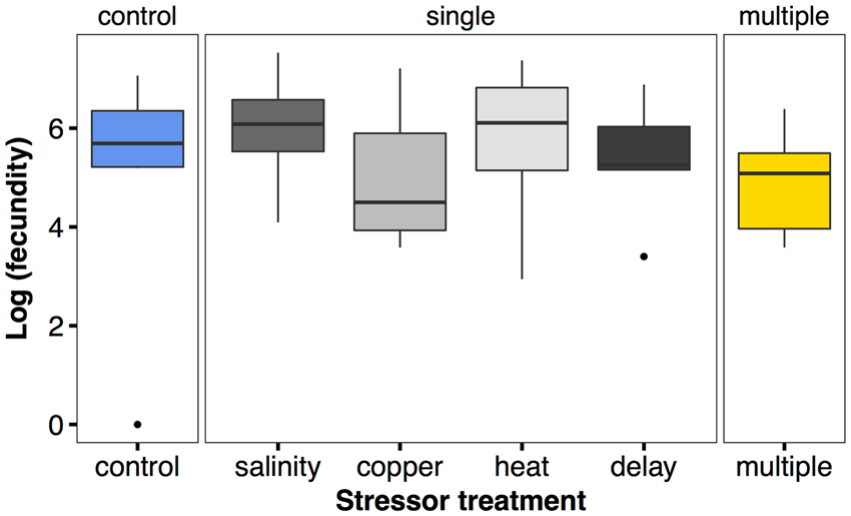
Comparison of control (blue), single stressor (grey), and multiple stressor (yellow) effects on log (fecundity). Boxplots from raw data, treatment effects on x-axis, fecundity on y-axis.

While stressor treatments did affect log fecundity (Fig. 3, Tables 2 and 3), the effect was surprising: colonies in the heat single stressor treatment outperformed colonies in the control treatment, in the copper and salinity treatments there was a slight tendency for better performance, and in the delay and multiple stressor treatments performance was not significantly different from the control (Table 3).

## Discussion

After treating *Bugula* settlers to stressor levels as found at field sites, and then transplanting these settlers into a field site, we found treatment effects most evidently manifested in survival. Individuals in the control group survived best, those exposed to multiple stressors survived least. Those individuals/colonies that survived stressor treatments and were out in the field showed slight carry-over effects to later developmental stages, such that colonies that had experienced warmer temperatures as a single stressor produced slightly more offspring. This carry-over effect was not reflected in colony size, where we found no significant treatment effects. Against our expectation, our results are congruent with antagonistic effects of multiple stressors on survival: the decrease in survival under multiple stressors was lower than the null expectation under multiplicative stressor effects. The magnitude of stressor effects was context-dependent.

Conspecific density in the field positively correlated with the likelihood of survival: individuals that had experienced multiple stressors and had low conspecific density survived least. Our results show that the total impact of multiple stressors can be dependent on other environmental parameters, reinforcing previous findings that indicated stressor outcomes can depend on source habitat condition, food availability, or thermal environment^39, 40, 65^. We believe that facilitation or microenvironmental variation caused the positive density-dependence of survival in our field study. Facilitation occurs when conspecifics increase individual fitness by benefitting one another^66, 67^. Sessile marine invertebrates were often thought to be negatively density-dependent through competing for resources such as food^68^, space^69–71^, and oxygen^72^. For example, in the bryozoan *Electra pilosa*, the presence of neighbouring conspecifics can reduce colony growth^73^. However, we now know that positive density-dependence or facilitation is also common^74–76^. In another bryozoan species, *Watersipora subtorquata*, facilitation was most likely when resources were experimentally elevated^77^. Because we measured survival during early succession when space was abundant and competition low – constituting a high-resource environment – facilitation effects were likely. Microenvironmental variation, however, is an equally plausible driver of the positive correlation between conspecific density and survival. Conditions at the field site can vary over small spatial scales, thereby strongly affecting fitness parameters^78, 79^. Patchy resource distribution can easily in- or decrease overall survival within small areas (such as a 11 cm * 11 cm plate, within which conspecific density was calculated), thereby creating a correlation between focal individual survival and conspecific density^80, 81^.

Exposure to multiple stressors during early life history mostly manifested in survival, slightly in fecundity and not all in colony size in later life history. This is not uncommon, as many stressors only have immediate effects on exposed individuals^82–85^. For instance, in a gastropod, the effects of larval exposure to salinity and a pollutant reduced survival but had little effect on later life history stages^83, 86^. Similarly, in *Bugula* salinity or copper exposure decrease growth rates or survival, but after exposure and transplantation into the field this effect subsides^82^. However, in contrast to our and Piola and Johnston’s^83^ findings, another study on *Bugula* found larval exposure to 100 µg/L copper or more to decrease survival long after exposure, albeit with large spatial variation in this effect^87^. In our study, single and multiple stressors decreased survival, with multiple stressors exerting the strongest effects. Later in individual development treatment effects manifested slightly in fecundity, but not in colony size. It is plausible that stressor responses manifesting in fecundity but not colony size is an artefact; fecundity could only be estimated for a subset of temporal blocks in which most individuals reached sexual maturity (colony size and survival were measured at week 4, but fecundity was measured at week 11). Our results on colony size are hence better replicated and hence more reliable. Overall, we think it is most likely that stressor exposure had strong effects on early survival in *Bugula*, but only few on subsequent fecundity and growth.

We proceed cautiously with our interpretation of potential synergistic or antagonistic effects, as the testing of interaction effects requires a full factorial design. This was not feasible for four different factors. Rather than having low levels of replication at each of the necessary 16 treatment levels, we wanted to achieve solid temporal and spatial replication. Instead of testing for interaction effects using ANOVA, we hence computed a null expectation under multiplicative effects and compared this with our result. Our results are congruent antagonistic stressor effects. Antagonism here means the effect of multiple stressors is smaller than the summed effect of each individual stressor^35, 61^, but not necessarily to a degree where stressor effects completely cancel each other out.

Interactive stressor effects are common and we will here examine some of the known single and combined stressor effects on benthic marine invertebrates. Copper and temperature stress often elicit a synergistic response (e.g., in oysters^32^). Similarly, synergistic responses are common for copper and salinity stress (e.g., in oysters^32^ and crabs^34^). Copper toxicity increasing with decreasing salinity has been attributed to osmoregulation being affected by heavy metals^88^, but other mechanisms also appear likely^31^. Temperature and salinity stress can elicit both synergistic and antagonistic effects in exposed organisms^5, 7^. One study is particularly noteworthy in this context: when simultaneously testing for the effects low salinity, elevated temperature and copper in an oyster, synergism was detected^32^. We found no studies investigating the effects of prolonged larval durations combined with other stressors. In *Bugula*, which has a non-feeding larval stage, prolonged larval delay depletes energy reserves and decreases fitness in later life history stages^48, 89^. Because tolerance to stress is energy-limited^12, 40^, we expected additive or synergistic effects when energy-depleted individuals face additional stressors. Therefore, previous findings on stressor responses seem to point to a synergistic response under exposure to low salinity, elevated temperature, heavy metal pollution and prolonged larval duration, yet our finding points to antagonistic or additive effects. Many explanations for this finding are likely. For example, under co- or cross-tolerance to stressors, individuals or species that are resistant to one stressor are also resistant to another^8, 90^. Co-tolerance to stressors is common^8, 91–94^ and implies that individuals that are sensitive to one stressor will be sensitive to another. Because viability within populations is rarely equally distributed^95^, there will always be individuals more sensitive than others. Stressors then remove the most sensitive individuals from a population. Indeed, we did find elevated fecundity in some of the single stressor treatments – perhaps this was a result of selection against the least viable individuals in our cohorts.

While our results suggest antagonistic stressor effects of four co-occuring stressors on survival, recent findings suggest that synergisms are predominant under multiple stressors, especially when more than two stressors are tested^5^. Indeed, we had expected to find synergism. Gunderson *et al*.^8^ recently proposed that when organisms are simultaneously exposed to stressors, stressor synergism is the most likely outcome because the increased stressor intensity is likely to overcome compensatory mechanisms. However, Coté *et al*.^35^ recently discovered in their meta-analysis and re-analysis of data that synergistic, additive or antagonistic are equally common under multiple stressors: synergisms have been overestimated due to lack of formal statistical testing. Furthermore, stressor levels tested may also affect stressor interaction effects^8^. Stressor levels are commonly adjusted to extreme future levels^7^. The reaction to shock treatments or very high stress levels often differs from relatively low levels of stress^8^, and we know from genetic and quantitative genetic analyses that there are different (metabolic) reactions to various stressor intensities^44, 96–98^. Gaining more fine-scaled stressor dose-response curves might elucidate this problem. We speculate that at high stressor levels synergism might be common, while at low stressor levels antagonistic stressor effects predominate.
